# 2433 Community forums as a channel for communicating with the public and to influence perceptions of cancer clinical trials

**DOI:** 10.1017/cts.2018.55

**Published:** 2018-11-21

**Authors:** Elizabeth Flood-Grady, Vaughan James, Janice L. Krieger

**Affiliations:** Clinical and Translational Science Institute, University of Florida

## Abstract

OBJECTIVES/SPECIFIC AIMS: Cancer clinical trials (CCTs) are vital tools in the advancement of cancer prevention and treatment. Yet, only 3%–5% of eligible patients enroll in CCTs. Low participation can be attributed, in part, to poor communication as well as a lack of awareness and understanding about CCTs. In order to increase participation in trials, interventions should foster meaningful communication about cancer prevention and CCTs, especially between medical professionals and members of the community. Community forums provide a channel to communicate about cancer with members public and to educate prospective participants about CCTs. Thus, our goal was to evaluate the efficacy of hosting community forums about cancer in order to educate the public and influence perceptions of CCT participation. METHODS/STUDY POPULATION: During the Spring of 2016, participants (n=51) who attended a community forum about CCTs completed a pretest and post-test survey assessing their understanding and perceptions of CCTs. RESULTS/ANTICIPATED RESULTS: Results from the pretest to post-test survey revealed a significant positive increase (*p*=0.01) in participants’ attitudes toward cancer clinical research as well as marginally significant increases in participants’ perceived subjective norms (*p*=0.06) about participating in CCTs and the perceived personal relevance (*p*=0.06) of clinical research participation pretest and post-test. DISCUSSION/SIGNIFICANCE OF IMPACT: Findings suggest that community forums about cancer and CCTs could lead to an increased awareness and understanding of CCTs among members of the population and could be useful channels for cancer interventions.

